# Scaffolding learning: From specific to generic with large language models

**DOI:** 10.1371/journal.pone.0310409

**Published:** 2024-09-20

**Authors:** David S. Yin, Xiaoxin Yin

**Affiliations:** 1 Lynbrook High School, San Jose, CA, United States of America; 2 Airbnb, San Francisco, CA, United States of America; Universiteit Gent, BELGIUM

## Abstract

Large language models such as ChatGPT have been shown to excel in solving complex math problems. However, they cannot solve basic arithmetic problems such as 758*639 = 484,362. This makes us ponder if LLMs have been trained to solve math and science problems in the right way. When a student learns math at school, she or he starts with arithmetic, then moves to word problems, polynomials, and calculus. Each skill she or he acquires will be used in the next stage to solve more advanced problems. In this paper we propose *Scaffolding Learning* for LLMs, which imitates how a student learns a subject in a step-by-step manner. For example, we first train an LLM to perform highly specific operations such as multiplication and division, and then apply such “skills” in a more generic task such as solving word problems. This is related to *Curriculum Training*, which trains a model on tasks following a specific order, such as training on easy tasks first and then gradually increases the difficulty. Our proposed approach goes from specific tasks to generic ones, which can be considered as a special case of Curriculum Training. Our empirical studies show that when an LLM has “mastered” a specific skill, only a small amount of training is required to teach it to apply the skill to a more generic application.

## 1. Introduction

In the past few years there have been various studies showing that large language models (LLMs) can be trained to solve various types of math problems, including grade school word problems and generic math problems from arithmetic to calculus. Today’s most powerful models such as GPT-4 and Bard can even solve some complex problems in Math Olympiads. However, even the most powerful LLMs often fail to solve some basic math problems in grade schools, such as multiplication and division with three or more digits. Here are some examples (Tested on July 25, 2024. GPT-4 uses Python code to answer arithmetic questions. In order to test the language model, one has to use OpenAI’s API.)

Ex. 1 758 * 639 (correct answer is 484362)

GPT-4o: The product of 758 and 239 is 181,162. (Incorrect)

We can see that GPT-4o fails to produce the right answer, probably because it has not seen this problem in its training data. Thus we try to teach it calculate step by step with an example. Here is our prompt:

Ex. 2 *Prompt: Example Question*: 237 * 453

*Example Answer: Decompose* 453 = 400 + 50 + 3. *Therefore*, 237 * 453 = 237 * 400 + 237 * 50 + 237 * 3 = 94800 + 11850 + 711 = 107361

*Question*: 7518 * 639(*correct answer 4804002*)

GPT-4o: To compute the product 7518 × 639, we can use the distributive property, breaking down 639:

639 = 600 + 30 + 9

So,

7518 × 639 = 7518 × (600 + 30 + 9) = 7518 × 600 + 7518 × 30 + 7518 × 9

Now we compute each term separately:

1. 7518 × 600 : 7518 × 600 = 7518 × 6 × 100 = 45108 × 100 = 4510800

2. 7518 × 30 : 7518 × 30 = 7518 × 3 × 10 = 22554 × 10 = 225540

3. 7518 × 9 : 7518 × 9 = 67662

Adding these together:

4510800 + 225540 + 67662 = 4803902

Thus, 7518 × 639 = 4803902

From the above examples, we can see that GPT-4o cannot perform some basic math operations such as multiplication of several digits. This will prevent it from solving more complex math problems involving such operations, just like how a student unable to do multiplication will likely get bad grades in math tests. It is surprising to us that such a powerful model which can solve advanced math problems such as differential equations fails on such basic operations.

This makes us ponder whether LLMs have been trained to learn math in the right way. When children learn math at school, they start with arithmetic, which enables them to perform basic math operations such as multiplication and division. Then they learn how to solve equations, and how to solve word problems. All the arithmetic skills they acquired before enable them to solve more advanced problems correctly.

Here is an example word problem: “There are 346 employees in a company and the average compensation per person was 4,328 dollars in the last month. What was the total compensation in this company last month?”. This is an easy problem for a sixth-grader, who has the necessary arithmetic skills. But this is extremely hard for ChatGPT because it has not learned how to perform multiplication. All it can do is try to remember previous results, which is not very useful in this situation.

In contrast to how a student learns math, LLMs have been trained in a very different way in previous works. As in [1], LLMs were trained with math problems of all levels, together with solutions. LLMs trained in this way successfully solved many hard math problems, including some Math Olympiad problems that most high school students cannot solve. However, they constantly fail in basic math operations that a middle schooler can solve.

This makes us think if we should train LLMs in a way that is more similar to how we teach students. In this study we first train our LLM to perform basic math operations, just like how an elementary school teacher would teach her students. Then we train our LLM how to use such basic operations in word problems, again similar to what a teacher would do. We found that after learning the basic math operations, our LLM quickly learns how to apply such operations in word problems, which enables it to solve these problems with very high accuracy.

This is only an example that illustrates how we can teach LLMs in a level-by-level manner, which is often called “Scaffolding Learning” in education. Math is just one of the many subjects on which a student would learn level-by-level. In kinetics, a student first learns the force analysis and Newton’s laws, before she or he is asked to solve kinetics problems. In calculus, a student first learns how to compute first derivatives, and then how to compute second derivatives and integrals, before they are asked to solve real-world problems. Although we only focus on basic math in this study, the same methodology can be applied to every field where a student learns in gradual advancements.

This is related to *Curriculum Learning* [2, 3], which trains a model on tasks following a meaningful order. The most common order is from easy to difficult, i.e., first trains a model on an easy task and then gradually increases the difficulty of tasks. For example, in [2] the authors trained a model to classify shapes, which starts from the easiest task of classifying single simple shapes, and gradually increases complexity by varying positions, rotating, adding multiple shapes, etc. Curriculum Learning has been widely used in many applications such as object detection [4], face detection [5], and machine translation [6]. Our proposed method, Scaffolding Learning, can be considered as a special case of Curriculum Learning. Scaffolding Learning goes from specific to generic, by first training a model on specific tasks such as multiplication and division, and then fine-tuning it to accomplish more generic tasks such as solving word problems.

Both Scaffolding Learning and Curriculum Learning can be considered as special cases of the *Pre-training and Fine-tuning Paradigm*. In the past decade many large models are first trained on a generic dataset (such as ImageNet [7] or CommonCrawl), and then fine-tuned on different specific tasks to solve specific problems. What Scaffolding Learning does is analogical to a student acquiring a specific skill in one course (e.g., arithmetic skill) and then learning to apply the skill on more generic problems (e.g., word problems).

Here are our main contributions in this paper:

- We propose Scaffolding Learning for LLMs, which first train an LLM on a specific problem, and then train it on a more generic problem using the specific “skill” learned.
- We show that Scaffolding Learning is highly effective, as an LLM can quickly learn to apply an acquired “skill” to a more generic problem.
- We propose a way to teach an LLM to perform arithmetic operations (addition, subtraction, multiplication and division) of many digits.

## 2. Related work

Before the era of deep learning, researches have attempted to solve generic math problems (such as word problems) in various ways. In [8] the authors categorize every important word in a problem into either a variable holder(container), a variable(quantity), an item(entity), or a change in the quantity of a variable(state transition). With this information, the problem can be solved by using it to create an equation.

WordproblemJohn(container)has3(quantity)apples(entity).Adamgave(statetransition)him(container)2(quantity)apples(entity).Howmanyapples(entity)doesJohn(container)havenow?Equation3+2=5

Another method of interpreting a word problem is equation mapping [9], which first maps each number in the problem to a variable, then uses a model to classify the problem into several equation templates.

In recent years deep learning has been applied to a variety of math problems, such as grade school math problems [9–11], symbolic regression which infers the formula behind a curve [12, 13], and differential equations [14].

After GPT gained popularity in recent years, it has been applied to solve math problems with Chain-of-Thought [15], Tree-of-thoughts [16], and Graph-of-thoughts [17], which construct the prompt with different structures and are shown to outperform simple prompts. In many such studies [18, 19] GPT has been used to solve various reasoning problems, especially math word problems. This illustrates the math capabilities and reasoning power of LLMs.

### 2.1 Scaffolding Learning vs. Curriculum Learning

*Curriculum Learning* was proposed in 2009 [2, 3], which trains a model on tasks following a meaningful order. The most common order is from easy to difficult, which first trains a model on easy tasks and then gradually increases the difficulty of tasks. For example, in [2] the authors trained a model to classify handwritten digits, which starts from clean digits and then moves to noisy ones and distorted ones. Curriculum Learning has been widely used in many applications such as object detection [4], face detection [5], and machine translation [6].

Unlike Curriculum Learning which usually goes from easy tasks to difficult ones, Scaffolding Learning goes from specific to generic, by first training a model on specific tasks and then fine-tuning it to perform more generic tasks. For example, in order to teach an LLM to solve elementary school math problems, we first fine-tune an LLM to learn how to perform arithmetic operations, and then it only takes a very small number of examples (e.g., a few hundred) to fine-tune it to solve word problems containing arithmetic operations, which the original model could not solve.

#### 2.2 Scaffolding Learning vs. pre-training

Scaffolding learning is used in nearly every education system because it is intuitive and effective. It allows a student to use her understanding of simple and specific concepts to understand more complex and general concepts. In this paper we apply the same philosophy to train deep learning models.

Scaffolding learning for deep learning shares some similarity with the pre-training & fine-tuning paradigm, such as the many studies on specific tasks using fine-tuned BERT [20]. A model could be pre-trained on a huge dataset such as ImageNet [7] or CommonCrawl, in order to give it a generic understanding of the domain. Then the model can be fine-tuned on specific tasks such as detecting inappropriate languages.

Unlike pre-training & fine-tuning which goes from generic to specifc, Scaffolding learning goes from specific to generic. It first trains the model on a specific task like arithmetic operations, then uses it to solve a broader, more generic task like word problems. Just like a student acquires one or a few specific skills in each course at school, and then learns to apply such skills on more generic problems, we are trying to train LLMs in that manner.

Scaffolding learning allows us to “teach” an LLM to perform a specific task that it was not able to do. Afterwards the LLM can combine its language understanding capability with the newly acquired skill to solve more generic problems.

## 3. Model training

We choose to use the Pythia-6.9B [21] as our base model due to its high performance [22] and open-source nature. In the following subsections we describe how we create the training data for two scaffolding learning tasks:

- Train an LLM to perform arithmetic operations, and then to solve word problems using the “arithmetic skills”.
- Train an LLM to derive the first derivative of a function, and then to derive second derivatives using the “first-derivative skill”.

### 3.1 Arithmetic operations

Our dataset is comprised of two parts. The first is a set problems of additions, subtractions, multiplications and divisions, with 100K problems of each type. Each operand is randomly sampled from a log-uniform distribution in range [10, 10000]. In subtraction and division the second operand is smaller than the first.

Most LLMs (including GPT-4) fail to calculate basic Arithmetic problems such as 758*639. Therefore, we try to teach our model to do the calculation like a human, in a *digit-by-digit* manner. For multiplication, we will be using a method that splits the problem into several single-digit sub-problems. For division we will use a process that splits the dividend into several chunks of digits that are easier to divide.

For these to be effective, we split each number into individual digits by inserting spaces between digits. Otherwise BPE (Byte-Pair Encoding) [23] would break a number into chunks with various lengths, and it is difficult for an LLM to even remember a multiplication table of such chunks. For example, when asked to solve the question 1009 + 8432, if BPE is used and the original question is tokenized into “100”, “9”, “+”, “84”, “32”, the model will probably get trouble aligning the two operands.

In this subsection we will describe how we convert a vertical arrangement of an arithmetic operation into a sequence of tokens that can be learned by an LLM. We will start from addition and subtraction and then move to multiplication and division.

#### 3.1.1 Addition and subtraction

For an addition problem, we simply break it into digits, so that each digit is a token for an LLM (as shown below).

Examplequestion3712+2401Exampleanswer3712+2401=3712+2401=6113

This is slightly different from an addition vertical as we do not indicate a carry. This is because the condition that a carry happens is rather simple (i.e., the digits sum to ten or above), and an LLM can easily learn that. Subtractions are handled similarly.

#### 3.1.2 Multiplication

Much more details are required for multiplication. If we simply use “7 5 9 * 8 4 2 = 6 3 9 0 7 8” as training data, the model will have a hard time figuring out how the answer is generated, and probably fails in a similar way as GPT does.

$$\begin{array}{l} Example\;of\;Vertical\;Multiplication\\ \begin{array}{c} 759\\ \underline{\times 842}\\ 1518\\ 3036\\ \underline{6072\;}\\ 639078 \end{array} \end{array}$$

Therefore, we try to convert the multiplication vertical into a sequence. Just like the vertical above, we first convert each row into a sequence. For example, the first row indicates “759 × 2 = 1518”, and we represent it with the following expression:

[(7*2=14)shifted2=1400+(5*2=10)shifted1=100+(9*2=18)=1518]=1518

This above row represents a multiplication between a 3-digit number and a single digit. “shifted x” means that the result should be shifted left by x digits. For example, “18 shifted 2” is 1800. In order to learn the above operation, an LLM would need to learn the following:

- A multiplication table for 1 through 9
- Shifting a number to the left by adding zeros at the end
- Adding multiple numbers represented by digits, such as “1 4 0 0 + 1 0 0 + 1 8 = 1 5 1 8”

We convert each row in a multiplication vertical as above, and we use different types of brackets (“<>”, “[]”, “{}”, “()”) to indicate the start and end of a scope. In the above vertical, the second and third row needs to be shifted, as follows:

[(7*4=28)shifted2=2800+(5*4=20)shifted1=200+(9*4=36)=3036]shifted1=30360

One difference between our representation and a typical multiplication vertical is that we calculate from left to right, instead of from right to left. In the above example, we first calculate 759 × 8 = 6072 (which then becomes 607200), and then 759 × 4 = 3036, and finally 759 × 2 = 1518. This is more consistent with the original order of the number, and looks more natural in the form of a sequence. Here is our full sequence for the above multiplication problem:

<[(7*8=56)shifted2=5600+(5*8=40)shifted1=400+(9*8=72)=6072]shifted2=607200>+<[(7*4=28)shifted2=2800+(5*4=20)shifted1=200+(9*4=36)=3036]shifted1=30360>+[(7*2=14)shifted2=1400+(5*2=10)shifted1=100+(9*2=18)=1518]=607200+30360+1518=637560+1518=639078

#### 3.1.3 Division

A different approach is used for division. We teach the model to do long division, which constantly subtracts the largest possible single-digit multiple of the divisor from the first few digits of the dividend. For example, for the division problem 615/47, our first step is to subtract (47 * 1) * 10 = 470 from the dividend and add 1 * 10 = 10 to the result. Here we do not break “47” into individual digits and then run the digit-wise multiplication as in Section 3.1.2, because that would require too many tokens for each multiplication and possibly exceed a model’s token limit. Instead, we rely on the model’s ability to “guess” the first digit of the quotient, which is the largest single-digit multiple of the divisor that is no larger than the first few digits of the dividend.

ExampleofLongDivision13R447)615¯47¯145141¯4

As one can see, the model would need to learn the following tasks to perform a division correctly: (1) The overall process of division, (2) whether a number is smaller than another, and (3) “guess” the largest single-digit multiple of the divisor that is no larger than the first few digits of the dividend. We will be focusing on making it understandable for the model. Below is an example of an expanded division operation.

Examplequestion615/47Exampleanswer615/47=615/47=<[{(61/47#because1*47&61|1,remainder=61-1*47=61-47=14)shifted1=10}+{(14shifted1+5=145/47#because3*47&145|3,remainder=145-3*47=145-141=4)=3}]=10+3=13>=13remainder4

We use single-character symbols for separators and operators, in order to lower the number of tokens needed. As an example, the “&” token represents “<=” (which is considered to be two tokens by our model).

### 3.2 Word problems

We use SVAMP [24] as our word problem dataset, which consists of 7,000 grade school word problems. The constants in each problem of this dataset can also be customized, allowing us to easily change the difficulty of problems.

In order to teach a model to apply the arithmetic operations learned in Section 3.1 to solve word problems, we expand the math operation in a word problem using the methods in Section 3.1, which makes our model apply its “arithmetic skills” in solving the word problems. For example, this is a word problems with expanded math operation:

OriginalquestionSamhas615emails.Hetakes194secondstoreadeachemail.Hesendsoneemailevery503seconds.Howlongwillittakehimtoreadallemails?ModifiedquestionandanswerSamhas615emails.Hetakes194secondstoreadeachemail.Hesendsoneemaileverysomeseconds.Howlongwillittakehimtoreadallemails?615*194=〈[(6*1=6)shifted2=600+(1*1=1)shifted1=10+(5*1=5)=615]shifted2=61500〉+〈[(6*9=54)shifted2=5400+(1*9=9)shifted1=90+(5*9=45)=5535]shifted1=55350〉+[(6*4=24)shifted2=2400+(1*4=4)shifted1=40+(5*4=20)=2460]=61500+55350+2460=116850+2460=119310(Note:Wehaveremovedthespacesbetweendigitsforbetterreadability.)

In this study we focus on integer operations, in order to simulate how an elementary school student learns math. Therefore, all constants in our word problems are rounded up and then verified for correctness. All problems that become invalid are filtered out. 2,217 problems remain correct and are kept.

When customizing the constants in each word problem, each original constant is first multiplied by a random number sampled from a log-uniform distribution of range [1000, 10000], and then constantly halved until it is less than 10000. This gives us a diverse range of constants that are usually consistent with the originals.

### 3.3 Derivatives

In addition to arithmetic operations and word problems, we also test Scaffolding Learning in computing the first and second derivatives of polynomial functions. It is shown that after an LLM is trained to compute the first derivatives, it only requires a very small number of training examples to teach it to compute second derivatives.

We use the derivatives section of DeepMind’s mathematics dataset [25] to automatically generate functions and their derivatives. When customizing the constants in the functions, we use values randomly sampled from a log-uniform distribution of range [1, 100].

The training data for first derivatives simply include the functions and their derivatives. For second derivatives, we include a very short rationale explaining that it is the first derivative done twice, as shown in the following example:

QuestionWhatisthesecondderivativeof62*k**2+43k-9?AnswerDerivativeof62*k**2+43k-9=124k+43.Derivativeof124k+43=124

## 4. Experiments

We used the Pythia-6.9B model [21] due to its high performance and open-source nature. The model was pre-trained on the Pile dataset [26] and can be downloaded on hugging face (https://huggingface.co/EleutherAI/pythia-6.9b). Example code of fine-tuning this model can be found at github (https://github.com/databrickslabs/dolly).

All our experiments were done using a computer with two NVIDIA A6000 GPUs, an Intel i7-12700K CPU, with Ubuntu 18.04 and Pytorch 2.0.0. When training models, we used DeepSpeed [27] to utilize multiple GPUs, and ZeRO [28] stage 3.

### 4.1 Arithmetic operations

To train our LLM to perform arithmetic operations, we generate examples using the method described in Section 3.1, and fine-tuned the pre-trained Pythia-6.9B model with the examples. An example is never fed to our model twice, which eliminates the risk of overfitting. The training set contains 450K examples, with equal numbers of addition, subtraction, multiplication, and division. The evaluation set contains 500 examples generated in the same way.

**Fig 1:**
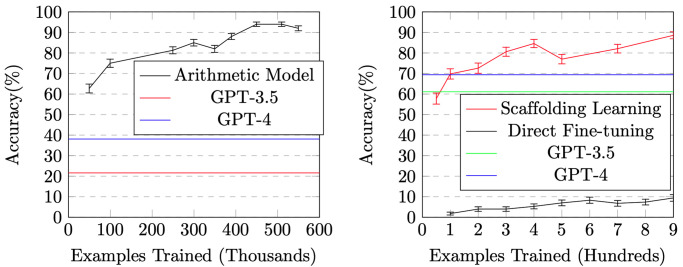
The effectiveness of Scaffolding Learning in applying arithmetic skills to word problems. The error bars represent standard deviations.

Fig 1A shows the evaluation accuracy vs. training examples for arithmetic operations. One can see that it takes hundreds of thousands of training examples for the model to learn how to perform arithmetic operations, with the accuracy growing from 75% with 100K training examples to 94% with 450K examples.

We submit each problem to GPT-3.5 (a.k.a. ChatGPT) and GPT-4 using OpenAI’s API. As shown in Fig 1A, their accuracies are much lower than our trained model.

### 4.2 Word problems

After fine-tuning the Pythia model to perform arithmetic operations, we fine-tune it to solve word problems as described in Section 3.2, where the training data contain the same expanded procedure of arithmetic operations as in Section 3.1. We want to see that after our model has mastered arithmetic operations, how quickly it can learn to apply such operations to solve word problems. We use a modified version of the SVAMP dataset (see Section 3.2 for details). After removing invalid cases from SVAMP dataset (such as those becoming invalid after rounding), we have 2,217 problems left. We randomly split them into a training set of 1,892 problems (85% of the dataset), and an evaluation set of 325 problems.

In order to test the effectiveness of Scaffolding Learning, we compare two approaches: (1) Scaffolding Learning, which uses the model fine-tuned on the arithmetic dataset (as in Section 4.1), and fine-tunes it on word problem training set. (2) Directly fine-tuning the open-source pre-trained Pythia-6.9b model on the same word problem training set.

The results are shown in Fig 1B. We can see that when the model has acquired “arithmetic skills”, it achieves 70% accuracy with only 100 training examples, and achieves almost 90% accuracy with less than 1,000 examples. In contrast, the directly fine-tuned model only has about 10% accuracy after one epoch of training, even the training data contains the full procedure of each arithmetic operation as described in Section 3.1.

This experiment shows that Scaffolding Learning is highly effective. Just like a student who has mastered arithmetic skills can learn to apply such skills to solve word problems with a very small number of examples, an LLM can do that same. When an LLM has been trained with hundreds of thousands of examples to perform arithmetic operations, it only takes tens or a few hundred examples to learn how to use its arithmetic skills to solve word problems. This is also because the LLM has always been able to understand natural language, just like a student.

As a summary, when a model is equipped with both language skills and arithmetic skills, it only takes tens or a few hundred examples to learn how to combine them to solve word problems.

### 4.3 Derivatives

We also use Scaffolding Learning to compute high-order derivatives with a very similar procedure to word problems: The LLM is first fine-tuned to compute first derivatives. Then it can learn to compute second derivatives with a very small number of examples. We use the derivatives section of DeepMind’s mathematics data generator to generate training and testing data, as described in Section 3.3.

**Fig 2:**
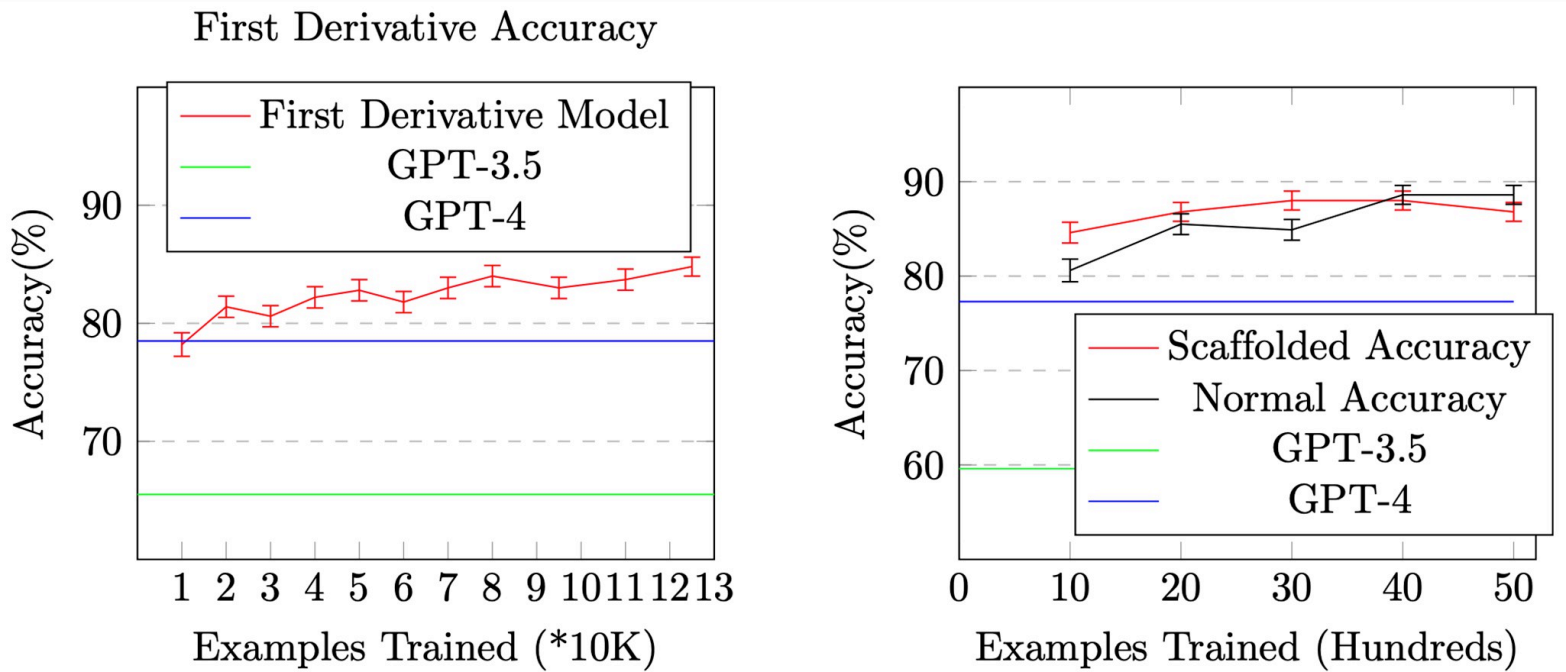
The effectiveness of Scaffolding Learning in applying the skill of computing first derivatives to compute second derivatives. The error bars represent standard deviations.

We first fine-tune a Pythia-6.9b model to compute first derivatives on a training set of 17,605 examples, and evaluate it on a test set of 1,784 examples. As shown in Fig 2A, the model achieves an accuracy of 85% with 125K training examples.

We then apply Scaffolding Learning, i.e., fine-tuning the above model to compute second derivatives on a training set of 10,000 second derivative examples generated by (Saxton et al. 2019). Again we compare it with directly fine-tuning Pythia model, on an test set of 1,081 examples. As shown in Fig 2B, Scaffolding Learning achieves higher accuracy after each number of training examples. To achieve about 85% of accuracy, it only requires 1,000 examples for Scaffolding Learning, and it takes 2,000 examples for direct training. This is a much smaller gap compared with arithmetic operations and word problems (in Fig 1B). This is probably because Pythia model has been trained on math dataset which includes polynomials and derivatives.

## 5. Discussions

Pre-training and fine-tuning has become the paradigm in creating Neural Networks and especially LLMs, which usually pre-trains on generic datasets (such as CommonCrawls and ImageNet) and then fine-tunes on specific tasks (such as question answering and face detection). Curriculum Learning is a special case of pre-training and fine-tuning, as it pre-trains a model on a meaningful order. The most common order is to train on an easy version of the task first, and then keeps fine-tuning the model on tasks with gradually increased difficulties. We propose Scaffolding Learning, which is a special case of Curriculum Learning. It first fine-tunes a base model on a specific task, in order to learn a particular “skill” such as arithmetic calculations. Then the model is fine-tuned to apply this skill on more generic problems.

In order to measure the effectiveness of Scaffolding Learning, we test how quickly a model can learn to apply an acquired skill to a new scenario. For example, we first fine-tune an LLM to enable it to perform arithmetic calculations, and then test how quickly it can learn to use its arithmetic skills to solve word problems. It turns out that an LLM can learn to apply its newly acquired skill with a surprising small number of examples (e.g., a few hundred). While if we directly fine-tune the LLM to solve word problems without first teaching it to perform arithmetic operations, the LLM could not learn anything with thousands of examples.

### 5.1 Why is it useful?

In many real-world applications in science and engineering, the training examples are generated by humans and exist in limited quantities. For example, in physics problems one often need to use calculus or statistics to solve a real-world problem (such as calculating the orbits of celestial objects). It is often challenging to directly train an LLM to solve such problems, because of the lack of sufficient training data. On the other hand, it is often possible to generate infinitely many examples for the underlying mathematical or technical problem. For example, one can generate infinitely many problems for calculus, with randomized equations and math tools such as Matlab or SymPy.

Scaffolding Learning can help us train baseline models that master various skills for the underlying problems, such as calculus, polynomial operations, and matrix operations. One can usually generate infinite training data for such tasks, and train an LLM to master such skills. Then the model can be fine-tuned to apply such skills to solve real-world problems, which often need to be created manually from real-world scenarios. Since only a small number of examples are needed for the second step, Scaffolding Learning can greatly reduce the manual efforts required, and increase the model’s accuracy on the real-world problems.

### 5.2 Possible limitations

The first limitation of Scaffolding Learning is that it is only suitable for tasks where one can generate many training examples for an important sub-problem. For example, it is challenging to apply it on algorithm problems, as it is non-trivial to create many algorithm problems with solutions.

The second limitation is that, after the model is trained to master the underlying skill, it still needs to be fine-tuned to learn how to apply this skill on the original task. While this can be straightforward for word problems, formalizing a problem is sometimes a most challenging part in solving it. For example, in game theory and equilibrium theory, it is more challenging to formalize the problems mathematically than to solve them. Scaffolding Learning cannot assist with formalizing problems, which usually requires common sense.

Despite the above limitations, Scaffolding Learning can still be applied to many applications where training data is limited, but one can generate many training cases for the underlying problem. It is our future work to study how Scaffolding Learning can be combined with common sense, potentially expanding its applicability to a broader range of tasks.

## 6. Conclusions

In this paper we present Scaffolding Learning, in which a large language model is first trained on a specific task (such as arithmetic operations), and then fine-tuned on a more generic task (such as word problems). This is analogical to how a student learns at school, by first mastering arithmetic skills and then learning to combine such skills with her language skills and common sense to solve real-life problems.

Our experiments show that Scaffolding Learning can be successfully applied to training large language models. When a model has been pre-trained to understand language and fine-tuned to master a specific skill (e.g., arithmetic operations), it only takes a very small amount of training for it to apply the skills on more generic tasks such as solving word problems. This would enable us to train a large language models to learn various specific skills, and then combine those skills with its language capability and common sense in many applications.
